# Effects of Electrodes on the Switching Behavior of Strontium Titanate Nickelate Resistive Random Access Memory

**DOI:** 10.3390/ma8105374

**Published:** 2015-10-26

**Authors:** Ke-Jing Lee, Li-Wen Wang, Te-Kung Chiang, Yeong-Her Wang

**Affiliations:** 1 Department of Electrical Engineering, Institute of Microelectronics, Advanced Optoelectronic Technology Center, National Cheng-Kung University, Tainan 701, Taiwan; hugh2224@gmail.com; 2 Department of Electrical Engineering, National University of Kaohsiung, Kaohsiung 811, Taiwan; jk220052@gmail.com (L.-W.W.); tkchiang@nuk.edu.tw (T.-K.C.)

**Keywords:** sol-gel, resistive random access memory, strontium titanate nickelate

## Abstract

Strontium titanate nickelate (STN) thin films on indium tin oxide (ITO)/glass substrate were synthesized using the sol-gel method for resistive random access memory (RRAM) applications. Aluminum (Al), titanium (Ti), tungsten (W), gold (Au) and platinum (Pt) were used as top electrodes in the STN-based RRAM to probe the switching behavior. The bipolar resistive switching behavior of the set and reset voltages is in opposite bias in the Al/STN/ITO and Pt/STN/ITO RRAMs, which can be partly ascribed to the different work functions of top electrodes in the ITO. Analyses of the fitting results and temperature-dependent performances showed that the Al/STN/ITO switching was mainly attributed to the absorption/release of oxygen-based functional groups, whereas the Pt/STN/ITO switching can be associated with the diffusion of metal electrode ions. The Al/STN/ITO RRAM demonstrated a high resistance ratio of >10^6^ between the high-resistance state (HRS) and the low-resistance state (LRS), as well as a retention ability of >10^5^ s. Furthermore, the Pt/STN/ITO RRAM displayed a HRS/LRS resistance ratio of >10^3^ and a retention ability of >10^5^ s.

## 1. Introduction

Novel technologies for creating next-generation nonvolatile memory (NVM) have attracted extensive attention because conventional memory is already approaching its scaling limits. Resistive random access memory (RRAM) is one of the promising candidates for NVM applications because of its superior properties, such as reversible resistive switching, simple structure, small size, low power consumption, high operation speed, and low cost [1]. The development of RRAM has recently received increasing interest. Different materials with resistive switching characteristics, such as metal oxide [2], perovskite [3], polymer [4], or biomaterials [5], have been reported. Perovskite strontium titanate (STO) exerts a significant potential for RRAM applications; however, the switching performance remains unsatisfactory for the low current ON/OFF ratio [6]. Doping impurities, such as V and Nb, in STO prepared by a pulsed laser deposition or sol-gel deriving method have been proposed to improve the device performance [7,8,9]. The current ON/OFF ratio is still in the range of 10^3^ and can be further improved. Ni in STO can enlarge oxygen vacancy concentration and increase oxygen permeation fluxes [10]. The incorporation of Ni in the STO to form strontium titanate nickelate (SrTiNiO_3_, STN) can improve the film quality for the chelating effect of Ni with Ti ions. High-performance RRAM can then be expected by synthesizing STN thin films. STN prepared by the sol-gel method on indium tin oxide (ITO)/glass substrate with the top Al electrode may further enhance the current ON/OFF ratio [11]. In addition to the thin film, metal electrodes may be an alternative to improve the switching behavior of the RRAM, particularly the work function difference of top and bottom electrodes. Therefore, the switching behavior of metal electrodes is worth investigating.

In the current work, STN thin films prepared by the sol-gel method on ITO/glass substrate were synthesized for RRAM application. Different top electrode metals (*i.e.*, Al, Ti, W, Au and Pt) with various work functions and styles on the ITO bottom electrode were employed. The effects of different top electrode metals on metal/STN/ITO/glass structures were investigated. The interesting switching behavior (e.g., set voltage) was also observed. Probing the role of metals on the resistive switching behavior has become necessary. Compared with other metals, the STN with the Al top electrode presented excellent resistive switching properties with a small degradation of the current ratio between the low-resistance state (LRS) and high-resistance state (HRS). However, the Pt/STN/ITO/glass RRAM device, which exhibits a set voltage in different polarities compared with that of Al/STN/ITO/glass, can be developed. The set voltage in different polarities may be partly attributed to the different work functions of top electrodes. In addition, analyses of the fitting results and temperature-dependent performances showed that the switching mechanism of Al/STN/ITO was mainly ascribed to the absorption/release of oxygen-based functional groups, whereas the Pt/STN/ITO switching can be associated with the diffusion of the metal electrodes.

## 2. Results and Discussion

This work focuses on the effects of the top electrode on the switching behavior. All the STN thin films were prepared at the same conditions to ensure fair comparison. The layer thickness was characterized by a transmission electron microscope (TEM). The root-mean-square surface roughness of the STN thin film was measured to be approximately 3.7 nm by atomic force microscopy (AFM). A smoother surface can be achieved in STN than in that of STO thin films. The X-ray diffraction (XRD) patterns of the STN thin film deposited on the glass and ITO/glass substrates were determined. The peaks around 25° resulted from the glass substrate, and no other apparent diffraction peaks existed. These findings revealed that the STN thin film is amorphous.

**Figure 1:**
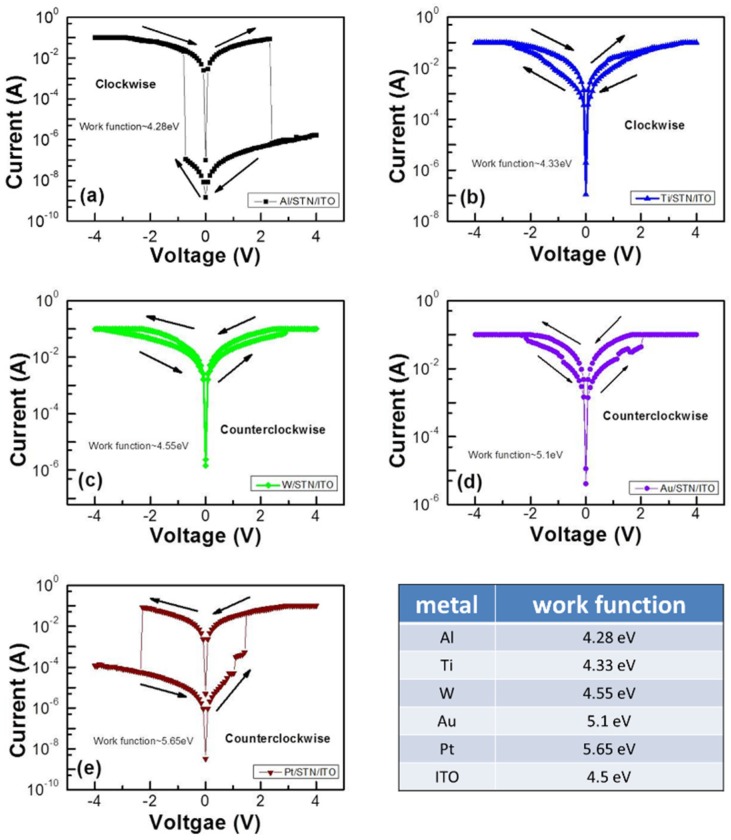
Typical switching current-voltage (I-V) curves of (**a**) Al/strontium titanate nickelate/indium tin oxide (Al/STN/ITO); (**b**) Ti/STN/ITO; (**c**) W/STN/ITO; (**d**) Au/STN/ITO; and (**e**) Pt/STN/ITO structures in bipolar resistive switching mode. The work functions of the used materials are also shown.

The typical current-voltage (I-V) characteristics of the RRAM devices with 50 nm-thick STN and different top electrodes (*i.e.*, Al, Ti, W, Au and Pt) are shown in Figure 1. The resistive switching curves were obtained from a positive voltage that reached +4 V and returned to 0 V, followed by a negative voltage that reached −4 V and returned to 0 V with 100 mA compliance current. No forming process is required in this switching behavior. The Al/STN/ITO RRAM device shown in Figure 1a demonstrated an excellent HRS/LRS resistance ratio of >10^6^ with set and reset voltages of −1.52 and 2.32 V, respectively. The switching behavior of the Ti/STN/ITO device with a HRS/LRS ratio of <10 is shown in Figure 1b. The set and reset voltages were −2.56 and 1.68 V, respectively. No clear switching behavior was observed in the Ni/STN/ITO structure, as shown in Figure 1c. The Au/STN/ITO structure with a HRS/LRS ratio of <10, set voltage of 2 V, and reset voltage of −2.16 V is shown in Figure 1d. The corresponding HRS/LRS ratio of 10^3^, set voltage of 1.52 V, and reset voltage of −2.24 V for the Pt/STN/ITO/glass RRAM is shown in Figure 1e. The switching behavior and polarity of the set/reset voltage largely depended on the electrodes. Evident resistive switching properties were only observed in the Al/STN/ITO and Pt/STN/ITO structures. Notably, the set and reset voltages of the Al and Pt top electrode structures are inversely related. This condition can be partly ascribed to the work function difference.

**Figure 2:**
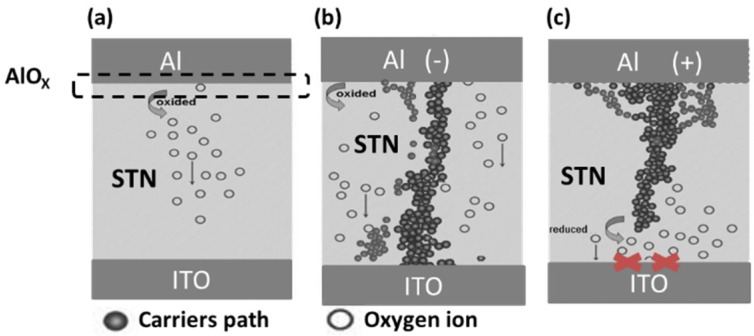
Possible resistive switching mechanism of the Al/STN/ITO device. (**a**) Thermal equilibrium; (**b**) Negative bias at the Al side; and (**c**) Positive bias at the Al side.

Al is recognized as one of the most oxidizable metals. Redox reaction occurs at the interfaces between the STN layer and Al electrode to form an Al oxide (AlO*_x_*) layer, which can help form conductive filament paths and lead to LRS, as shown in Figure 2b [12]. The conduction paths of the positive voltage to the Al electrode (Figure 2c) will be blocked, thus resulting in HRS.

**Figure 3:**
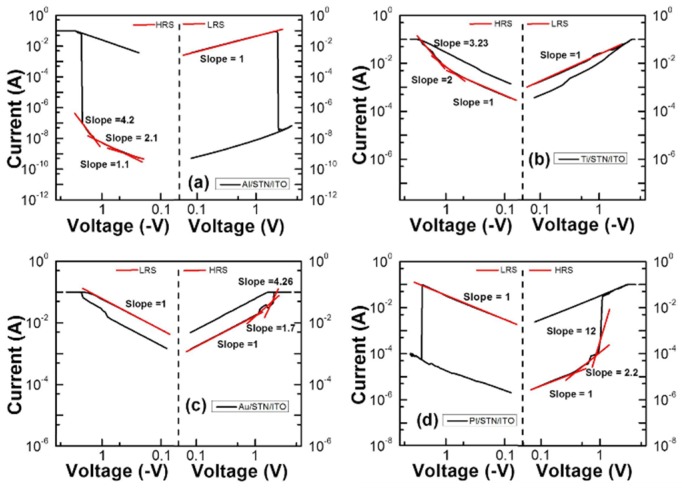
Fitting results of the top electrode/STN/ITO/glass structures in both high-resistance state (HRS) and low-resistance state (LRS) for (**a**) Al; (**b**) Ti; (**c**) Au; and (**d**) Pt.

The curve fittings by log I-log V [13] for HRS and LRS were performed, as shown in Figure 3, to clarify the mechanisms underlying the resistive switching characteristics of metal/STN/ITO RRAMs. The fitting results of LRS illustrate the linear current dependence on voltages, which correspond to the ohmic conduction behavior (slope ~1.0). The HRS conduction, in the form of the ohmic region (I ∝ V) and Child’s law region (I ∝ V^2^) [14], is dominated by the defect-conductive space charge-limited current mechanism. This scenario implies that the resistive switching mechanisms can be considered as the formation and disruption of local conducting paths.

**Figure 4:**
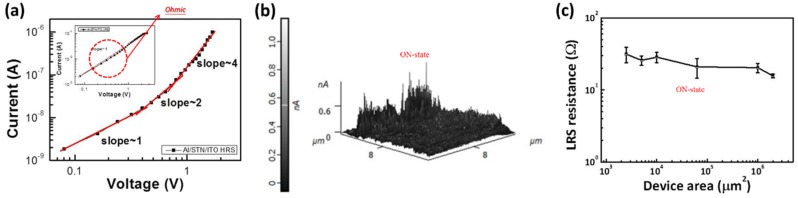
(**a**) Log I-V curve; (**b**) Current-sensing atomic force microscopy image devices in the LRS; and (**c**) area dependence of LRS resistance of STN memory device.

The resistive switching behavior of the STN memory device can be explained by a filament conduction mechanism as follows: (1) The I-V curve in the ON state can be fitted to a straight line with a slope of 1 in log-log scale, suggesting the ohmic characteristic, as shown in Figure 4a. The different conduction behavior in the ON and OFF states also implied that the high conductivity in the ON-state device is a localized conducting effect rather than a homogeneously distributed one. The formation of localized conductive filamentary paths was also confirmed by current-sensing atomic force microscopy (CS-AFM) characterizations, as shown in Figure 4b. (2) The influence of the device area on the resistance of the ON state is shown in Figure 4c. The LRS resistance related to the device area is insignificant. These results are similar to the report of Wong *et al.* [15], which supports the mechanism of the formation and rupture of conducting filaments. The weak cell-area dependence of the resistance in LRS showed that the set process matches the filamentary conducting mechanism [16].

The work function differences of Al, Ti and W between ITO are −0.22, −0.17 and 0.05 eV, respectively. The log I-V results indicated that the W/STN/ITO device exhibits approximately no memory properties. This result might be ascribed to the easy formation of filament in the large work function with differences between the top and bottom electrodes. The large work function difference between the top electrode and the bottom electrode (ITO) may then be beneficial for the resistive switching of the STN memory device. The different I-V curves of the Al/STN/ITO device compared to the Ti/STN/ITO device might be attributed to the larger work function difference (−0.22 eV) of Al, which also served as an oxygen-guttering material to induce the oxygen vacancies at the Al/STN interface, thereby modifying the oxygen vacancy distribution within the STN thin films and further enhancing the resistive switching properties [12]. These two factors may explain the better switching behavior of Al/STN/ITO than that of Ti/ATN/ITO.

The work function difference of Au and ITO is 0.6 eV (5.1–4.5 eV). A clearer switching behavior with a positive set voltage can be observed with the increasing work function difference. The work function difference of Pt and ITO is 1.15 eV (5.65–4.5 eV). The positive voltage on the Pt top electrodes generates a high electric field that drives metal Pt ions into the STN layer and forms conducting filaments inside the STN layer. The device reaches and then retains the LRS after the set process. The rupture of the conducting filaments resets the device. The HRS is finally attained again [17] when negative bias is applied to the Pt top electrode.

**Figure 5:**
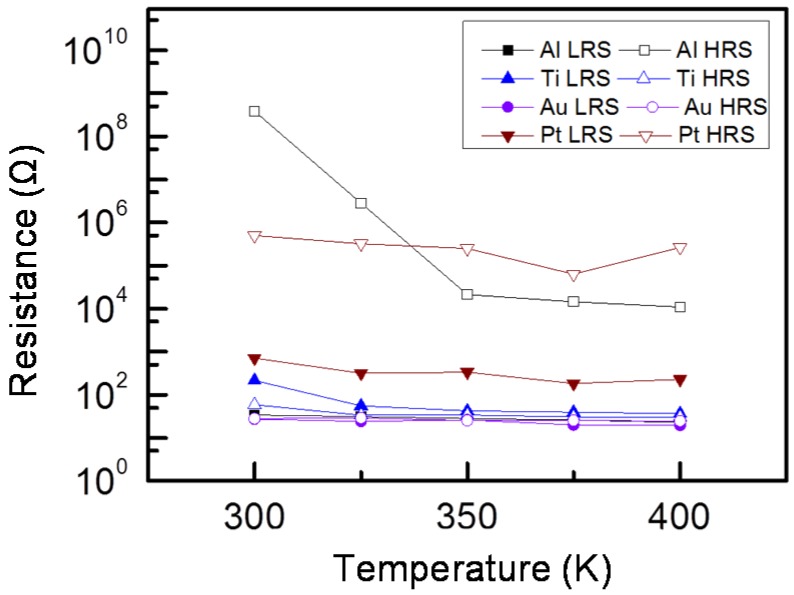
Temperature-dependent resistance of STN memory devices measured between 300 and 400 K.

The temperature effects of the HRS and LRS resistance are shown in Figure 5. The HRS resistance de-creases with the increasing temperature; the accumulated large number of oxygen vacancies modulates the conductivity of the oxide, which exhibits a typical semiconductor behavior [18]. By contrast, the LRS resistance evidently decreases with the decreasing temperature, which exhibits the feature of a metal conduction mechanism [19]. The resistance of the LRS in the Al/STN/ITO structure is inversely proportional to the temperature, whereas the resistance value increases with increasing temperature in the Pt/STN/ITO/glass memory device. These conditions indicate a semiconductor behavior and a weak metallic-like behavior. Accordingly, the switching mechanisms of the Al/STN/ITO/glass and Pt/STN/ITO/glass contributed to the migration of oxygen vacancies and formation of metal filaments, respectively.

**Figure 6:**
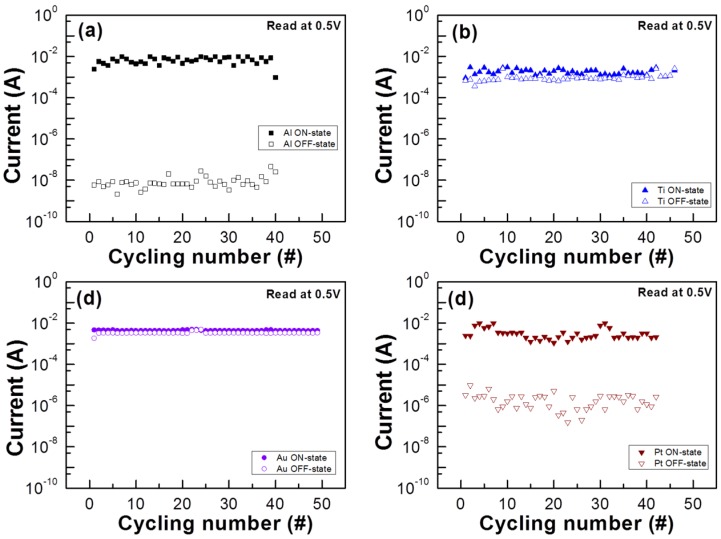
Endurance performance of the top electrode/STN/ITO structure with top electrode material of (**a**) Al; (**b**) Ti; (**c**) Au; and (**d**) Pt RRAM operated at room temperature.

The endurance properties with the direct current (DC) swept of the STN-based RRAM operated at room temperature are shown in Figure 6. The resistive switching behavior patterns are all stably repeated for over 40 cycles at a reading voltage of 0.5 V.

**Figure 7:**
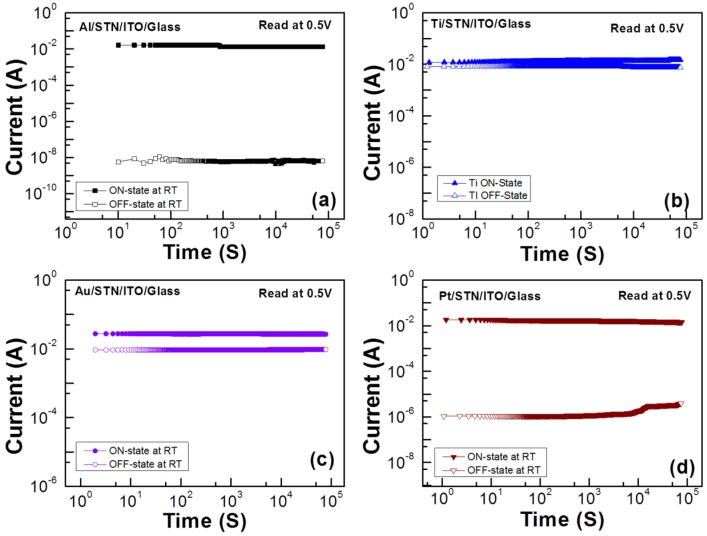
Retention test results of the RRAMs with the top electrode material of (**a**) Al; (**b**) Ti; (**c**) Au; and (**d**) Pt at room temperature (read at 0.5 V).

The retention test at room temperature under the bipolar resistive switching mode is shown in Figure 7. No serious degradation is observed after 10^5^ s. Consequently, the suitable non-volatility of the sample is demonstrated. This phenomenon indicates a reasonably stable behavior in terms of information storage capability.

Table 1 shows the performance comparison of the STN RRAMs with various top electrodes. The switching mechanism of the Al/STN/ITO RRAM can be described by the filament formation and rupture process as follows. The STN showed HRS state in the initial state. Given that Al is one of the most oxidizable metals, it attracts oxygen atoms from STN easily and forms an interfacial Al oxide layer [12]. When applying negative voltage on an Al electrode, the set process can occur. Some oxygen vacancies are filled by oxygen ions, which drift from Al to ITO. The electrons transport along the filament connected by oxygen vacancies, the connection causes the formation of conductive filament (CF) paths, and the device shows LRS. The reset process switching from ON to OFF states is performed by applying positive voltage on the Al electrode. When the oxygen ions drift from ITO to Al, the filament will be broken by oxygen vacancy re-oxidation [20].

**Table 1:** Comparison of metal/STN/ITO RRAMs with various top electrode metals. STN: Strontium titanate nickelate; ITO: Indium tin oxide; RRAM: Resistive random access memory; HRS: High-resistance state; LRS: Low-resistance state.

Metal	Al	Ti	Au	Pt
On/Off Ratio	10^6^	10	10	10^3^
Retention (s)	>10^5^	>10^5^	>10^5^	>10^5^
*V*set/*V*reset (V)	−1.52/2.32	−2.56/1.68	2/−2.16	1.52/−2.24
HRS Current (A)	5 × 10^−10^	3 × 10^−4^	1 × 10^−3^	9 × 10^−7^
LRS Current (A)	4 × 10^−2^	3 × 10^−2^	6 × 10^−2^	2 × 10^−2^

## 3. Experimental Section

**Figure 8:**
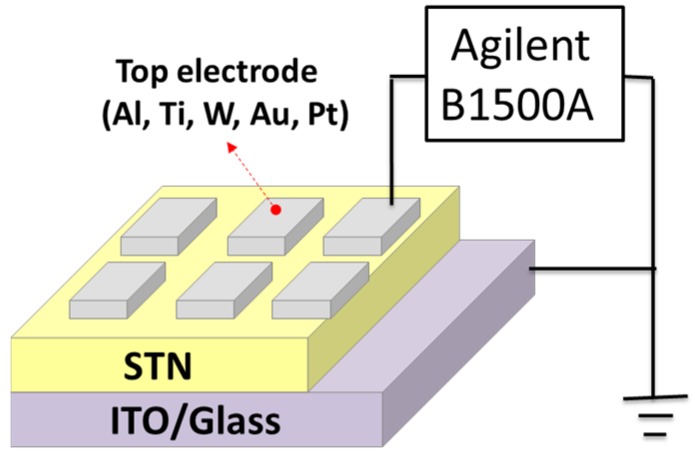
Device structure and measurement setup with Agilent B1500A.

The solution for STN thin films was prepared by sol-gel method. Strontium acetate (429 mg) was added into glacial acetic acid (4 mL). The mixture was stirred at 100 °C for 30 min to completely dissolve the strontium acetate powder solute into the solvent, which formed the clear solution A1. Titanium isopropoxide (0.61 mL) and nickel (II) acetylacetone (257 mg) were then mixed and completely dissolved with 2-methoxythanol (3 mL) to achieve solution A2. This mixture was subsequently added to acetylacetone (0.47 mL) and heated at 100 °C for 30 min while stirring until completely dissolved to produce the titanium-nickelate-based solution B. Solutions A1 and B were mixed, and 2-methoxythanol (1.54 mL) was successively added into the mixed solution. The mixture was stirred for 5 h to react thoroughly, thereby forming the final solution for the thin film deposition. The prepared 0.5 M precursor solution was spin-coated on the ITO/glass substrates at 5000 rpm for 20 s to form the STN films and then baked at 100 °C for 15 min. Finally, an 80 nm-thick metal top electrode was sputtered onto the deposited thin film by a radio-frequency magnetron sputter through a shadow mask to define the square-shaped electrodes. The electrode area measured 1.5 mm × 2 mm. The ITO, which serves as the bottom electrode, was grounded, and the bias was applied to the top electrode (*i.e.*, Al, Ti, W, Au or Pt). The device structure and measurement setup with Agilent B1500A are shown in Figure 8.

The STN thin film was characterized by X-ray diffraction (XRD), atomic force microscopy (AFM), and through the tapping mode of a scanning probe microscope. The electrical properties of the metal/STN/ITO memory devices were determined using a semiconductor analyzer (B1500A, Agilent Technologies, Santa Clara, CA, USA).

## 4. Conclusions

The effects of top electrode materials (*i.e.*, Al, Ti, W, Au and Pt) on the resistive switching characteristics of metal/STN/ITO structures have been investigated. The resistive switching mechanisms can be considered as the formation and disruption of local conducting paths. Metal work function can play an important role in the switching behavior. The set voltage changes from negative to positive with the work difference (relative to ITO) from negative to positive (−0.22 eV to 1.15 eV). A large work function difference can also be helpful for the switching behavior. This condition can be confirmed by the Al/STN/ITO and Pt//STN/ITO structures, not only in the resistive switching characteristics but also in the memory window. Given the stability and compatibility requirements for the STN process, the optimized work function of the top electrode material was also indicated. Finally, the points of optimized top electrode materials generated in this study can be applied to other RRAM materials in principle.
